# Urban Noise Pollution in Kumasi Metropolitan Assembly, Ghana: Implications for Public Health and Quality of Life

**DOI:** 10.1155/tswj/5769240

**Published:** 2025-08-19

**Authors:** Lyndon N. A. Sackey, Ebenezer E. Y. Amuah, Daniel K. O. Asamoah, Bernice Amoah, Brown C. Amoyaw, Benjamina A. Tettey

**Affiliations:** Department of Environmental Science, Kwame Nkrumah University of Science and Technology, Kumasi, Ghana

**Keywords:** Environmental Protection Agency (EPA), Kumasi Metropolitan Assembly (KMA), noise, noise dose, noise pollution, sub-metro

## Abstract

This study focused on urban noise pollution in the Kumasi Metropolitan Assembly, Ghana, examining its implications for public health and quality of life. Five submetropolitan areas: Subin, Bantama, Manhyia North, Manhyia South and Nhyiaeso were selected to represent different neighbourhood types: commercial, residential and mixed-use zones. Ambient noise levels were monitored using a JD-801A sound level meter. Generally, the noise level ranged between 51.86 and 82.87 dB. Manhyia South recorded the highest average noise, ranging from 58.65 to 82.87 dB, and Nhyiaeso recorded the lowest, ranging from 51.86 to 74.83 dB. Commercial areas had the highest noise levels due to overcrowded areas, traders and purchasers, deafening noise from public address systems, and overwhelming honking from vehicles. The study also revealed average noise levels across all five submetros significantly exceeded the WHO 2018 Environmental Noise Guidelines, posing a potential health threat, including cardiovascular diseases, sleep disruption and cognitive impairment. The findings emphasised the urgent need for regulatory enforcement, spatial noise planning and community-based noise mitigation strategies under the oversight of the EPA Ghana.

## 1. Introduction

The increased urbanization and industrialization in contemporary societies has led to a significant increase in noise pollution, a complex environmental issue with implications for public health and well-being [1]. As urban areas witness heightened human activities, including traffic, industrial operations and recreational pursuits, the acoustic landscape changes, leading to elevated noise levels [2]. The Ghana Environmental Protection Agency (EPA) is responsible for controlling the amount, intensity and quality of noise. The World Health Organization (WHO) states that prolonged noise exposure can negatively impact health, with road traffic being the primary source of ambient noise. In European cities, noise causes 587,000 disability-adjusted life years (DALYs) to be lost annually, with road traffic noise accounting for most of these losses [3]. Similar studies have also identified the significance of monitoring urban traffic noise in densely populated cities. For instance, Ranjan et al. [4] in their study *“Monitoring of traffic noise pollution in urban Patna, Bihar, India”* revealed that major intersections in Patna recorded noise levels well above the permissible limits, primarily due to uncontrolled vehicular flow, inadequate enforcement and infrastructural constraints.

The EPA in Ghana has established regulations regarding the amount of ambient noise acceptable in different locations [5]. Residential areas can have ambient noise levels of up to 55 decibels (dB) during the day and 48 dB at night. The acceptable noise levels for regions surrounding health and educational facilities are 50 dB at night and 55 dB during the day, respectively [6]. Light industrial or commercial activity areas have noise limits of 60 dB at night and 55 dB during the day [7].

The increasing noise in Kumasi can pose significant health risks to residents. This increasing noise can lead to reduced quality of life, increased healthcare costs and decreased property values [8]. The issue is expected to worsen as more people move into cities, affecting residents' overall well-being and satisfaction. This study can help local government agencies, urban planners and legislators create policies that promote peaceful and liveable urban environments, reducing the effects of noise pollution.

The aim of the study was to examine the sources, levels and effects of noise pollution on public health and quality of life. Specifically, the study attempted to (1) assess noise levels across the five submetros of the Kumasi Metropolitan Assembly (KMA) to determine the overall noise pollution level, (2) conduct a comprehensive survey and analysis of noise levels in various neighbourhoods and areas within KMA, and (3) assess the potential health impacts of urban noise pollution in the KMA.

## 2. Materials and Methods

### 2.1. Study Area

**Figure 1:**
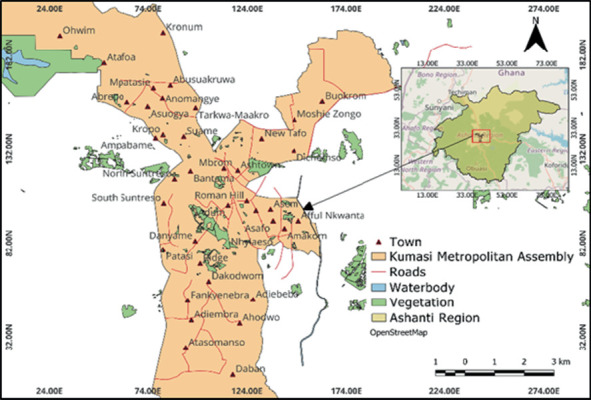
Map of Kumasi Metropolitan Assembly.

The study was conducted in some selected parts of the KMA which is located in the central part of the Ashanti region (Figure 1). Geographically, KMA is located at latitude 6° 41⁣′ 0⁣^″^ N and longitude 1° 37⁣′ 0⁣^″^ W [9]. It is a fast-growing metropolis with an estimated population of more than two million people and an annual growth rate of about 5.4% [10]. The metropolis is approximately 254 km^2^; its physical structure is circular, with a centrally located commercial area [11]. There are five (5) submetros under KMA which are Subin, Nhyiaeso, Bantama, Manhyia North and Manhyia South.

### 2.2. Sample Selection

The study sites were selected to represent different land uses in the KMA, including commercial, residential, and mixed-use areas. The criteria for site selection considered population density from the last census and land use classification (e.g., Central Business District, suburbs). Then, 30 sites in all submetros were specifically selected to maximise spatial coverage and variation.

### 2.3. Sample Method

A calibrated SLM with a 30–130 dB range and 3.5-dB precision was used to monitor noise levels in the various submetros [12]. ‘A' weighting was applied for measuring the sound level as it replicates the response of the human ear to noise, and the measuring unit is denoted as dB (A). Two (2) measurements were taken from four different points at each location, and the average noise level was obtained. Readings were taken from Monday to Friday for two weeks from 06:00 to 22:00 h. Measurements captured only daytime hours (6 AM–10 PM), on weekdays only. The study specifically targeted typical daytime noise conditions; nighttime/weekend data were excluded.

#### 2.3.1. Temperature

An electronic thermometer GM320S was used to accurately measure the temperature. The thermometer was placed between 1.2 and 2 m (4.1 and 6.5 ft) to gauge the temperature.

#### 2.3.2. Humidity

Relative humidity was measured using a digital hygrometer, ThermoPro TP49. For accurate readings, the device was held 5 ft (1.5 m) above the ground. Readings were taken to monitor stability.

#### 2.3.3. Elevation

A levelling instrument Laser Distance Meter P60L was used to collect elevation data and analysed with respect to noise levels at every location.

### 2.4. Mapping

A GPS device Essentials App was used to select coordinates at several study locations. The coordinates were converted into maps using ArcGIS. The locations of the commercial, residential, and mixed areas were depicted on a noise distribution map. A sufficient number of locations around the areas were chosen for taking noise level measurements, and these locations were highlighted on the map.

### 2.5. Potential Health Risk

#### 2.5.1. Noise Exposure Computation

Table 1 provides information on the environmental noise guidelines' recommended exposure limits strength for common environmental noise sources. It enables one to compare measured noise levels in the environment and the possible impact they could cause to human health.

### 2.6. Statistical Analysis

Statistical Package for Social Sciences (SPSS), Version 27.0, and Microsoft Excel 2010 was used to analyse the data and transform data for export to ArcGIS for the noise distribution mapping.

## 3. Results and Discussion

### 3.1. Submetro, Location and Time Analysis

Babisch [13] reported that commercial zones often go above permissible noise levels due to high traffic amounts and economic activities. From Table 2, commercial area, Adum, recorded the highest average noise level of 80.59 dB among all the selected neighbourhoods under the submetro in the afternoon as a result of overwhelming horns from cars, loud public address systems used by shops and overcrowded passersby, sellers and buyers. The average noise level of 80.59 dB for commercial surpasses the EPA standard of ambient noise (55 dB during the day). Bantama main market (Table 3) recorded the highest mean noise of 80.33 dB in the afternoon stemming from overcrowded people and passersby, excessive honking from motors and cars, loud corn mill machines from the market and buyers and sellers. The average noise of 80.33 dB in the afternoon exceeds EPA acceptable ambient noise standard (55 dB during the day). Commercial average noise level, 65.74 dB, was also recorded in the evening at Yenyawoso as a result of horns from trucks and cars in traffic and passersby with minimal buying and selling by the roadside (Table 4).

Commercial area, Santase, recorded the highest average noise level of 72.50 dB across all the selected neighbourhoods within the submetro, and it was a result of loud noise and horns from vehicles, passersby, conversations, distanced noise from public address system, and buying and selling from opened shops and stores.

The differences in noise levels between various kinds of areas (residential, mixed-use and commercial) follow patterns documented in studies by Moudon et al. [14] and Park and Evans [15] which show that commercial and mixed-use areas typically have higher noise levels due to their changing activities and density of residents.

The average noise level in Amakom's mixed area was 56.80 dB (Table 2) in the evening. This level was consistent with prior research findings, which showed that mixed-use areas frequently encounter moderate noise levels due to a combination of residential and commercial activity [16]. Kritzinger et al. [17] found similar evening noise levels in mixed-use zones in urban South Africa. The residential (middle) section in Amakom had a peak noise level of 61.28 dB (Table 2) in the afternoon. This level is slightly greater than that observed in several urban household studies. For example, Guski et al. [18] discovered that residential areas in European cities often had noise levels ranging from 50 to 55 dB during the day. The higher levels observed in Amakom were a result of the presence of motors and cars passing by and making loud noise, human conversations and a chop bar joint with a loud sound system, which was consistent with Bader et al. [19]'s findings that urban residential areas with nearby commercial activities tend to have elevated noise levels. The residential (low) area at Amakom had an average noise level of 64.08 dB in the evening. This level far exceeds the EPA's recommended ambient noise levels for residential areas (55 dB during the day). The elevated noise levels in residential (low) places are similar with the findings of Monroe [20], who discovered that lower income residential neighbourhoods frequently experience higher noise pollution due to their closeness to noise sources such as bars and food establishments. This is consistent with the study's findings of excessive noise from beer bars, conversations and trucks in the region. In the afternoon, the average noise level in Bohyen's mixed area was 80.23 dB (Table 3). This high level was caused by heavy car honking, pedestrian traffic and commercial activity. The noise levels in this location are with respect of results from other urban studies, which show that mixed-use zones with heavy traffic and commercial activity tend to have higher noise pollution. Kusumastuti and Nicholson [21] found that mixed-use zones frequently exceed standard ambient noise levels due to constant vehicular traffic and business activities. Residential (low) at Bohyen recorded an average noise of 60.94 dB (Table 3) in the afternoon as a consequence of a gutter construction producing loud and disruptive noise, and Alves et al. [22] established that noise from construction and urban infrastructure can cause residential noise levels to exceed recommended thresholds, affecting inhabitants' health and well-being. Hopkins and Reicher [23] found that public gatherings and events at places of worship can contribute to higher noise levels in nearby residential areas. This is aligned with the measured noise levels in Krofrom, where the church activities violated EPA rules and regulations (Table 4). The mixed area of Manhyia South (Table 5) recorded a mean noise level of 72.68 dB for evening. This high noise level in the mixed area was attributed to increased human activity, including passersby and honking, as suggested by previous studies establishing that traffic and pedestrian movement are key contributors to urban noise pollution [24, 25]. The mixed, Santase recorded the highest mean noise of 70.98 dB (Table 6) in the morning as a consequence of a preacher with an immoderate public address system, lorry traffic with few horns and few passersby. Gerike et al. [26] explored the impact of pedestrian activity on noise levels, bringing out that places with heavy foot traffic can increase noise levels due to discussions and other street-level interactions.

### 3.2. Potential Health Risk Based on WHO 2018 Environmental Noise Guidelines

Table 7 shows clear indications of increased noise exposure throughout the day, which extended to the morning, afternoon and evening hours. A comparison with the WHO Environmental Noise Guidelines 2018 (Table 1), which recommend a maximum value of 53 dB *L*_den_ and 45 dB *L*_night_ for road traffic noise with a strong recommendation, clearly shows that the measured values in all submetros significantly exceed the recommended limits.

Subin submetro recorded the highest noise levels of all the areas surveyed, averaging 70.10 dB in the morning, 72.27 dB in the afternoon and 65.69 dB in the evening. These values are 10–20 dB above the WHO limits. Manhyia South, Nhyiaeso and the other submetros also recorded noise levels above the recommended limits, indicating a widespread problem with excessive urban noise in the metropolis.

These consistently high ambient noise levels indicate a significant risk of chronic exposure, particularly from road traffic and other urban sources. According to the WHO [27], long-term exposure to such high noise levels is associated with a number of negative health effects, including an increased risk of cardiovascular diseases such as hypertension and ischaemic heart disease. Exceeding noise levels during evening and nighttime hours also raises concerns about sleep disturbance, which has a direct impact on general well-being and daytime performance [28]. Furthermore, prolonged exposure to high noise levels has been linked to cognitive impairment in children, especially in learning environments [29, 30]. Other associated effects include annoyance, stress and a diminished quality of life, all of which may contribute to long-term mental health challenges [31].

#### 3.2.1. Limitations

Our analysis did not include ISO/WHO annoyance surveys or community questionnaires. This omission means that our conclusions about the impact on quality of life are based on inference rather than direct measurement. Nighttime and weekend measurements were not taken, potentially underestimating cumulative exposure. Future work should integrate such tools to capture subjective quality-of-life impacts.

### 3.3. Association Between Ambient Noise Level, Humidity, Temperature and Elevation

Table 8 indicates the relation between ambient noise, temperature, humidity and elevation. Temperature, humidity and elevation had an impact on the changes in noise level within the submetros. Since the *p* value was smaller than the beta value, the beta value was statistically significant, meaning there was a correlation between the variables. Since the sig-value (0.00) was less than the beta value (0.956) for temperature, it was statistically significant, meaning temperature as a variable had an impact on the noise levels within the submetros.

For humidity, its sig-value (0.135) was greater than the beta value (0.085); so, it was not statistically significant, thereby not having an impact on ambient noise level. Elevation had an impact on noise level within the sub metros resulting from its statistically significant alpha (0.00) which was less than the beta value (0.175). Therefore, temperature and elevation had an impact on the noise levels within the submetros but humidity did not.

### 3.4. Ambient Noise Exposure Distribution Maps

Noise mapping used inverse distance weighting (IDW) interpolation in ArcGIS. A leave-one-out cross-validation was performed, yielding an RMSE of 4.09 dB, indicating moderate spatial uncertainty.

#### 3.4.1. Noise Exposure Distribution Map for Subin

**Figure 2:**
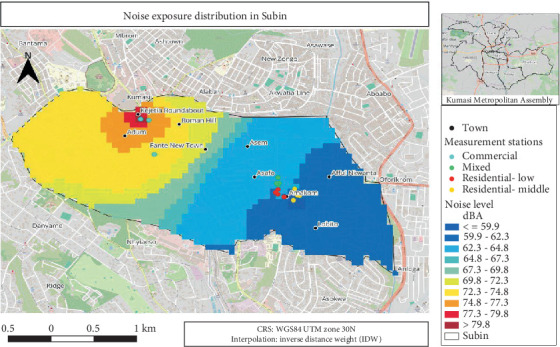
An average noise exposure distribution for Subin.

Subin area showed various noise levels in dB across different zones, using a colour gradient from blue (≤ 59.9 dB) to red (> 79.8 dB) (Figure 2). The highest noise levels were concentrated around Kejetia Roundabout and Adum (commercial areas), while the quieter zones are located in the southeastern areas like Amakom and Lobito. Measurement stations were marked for different land uses: commercial (sea blue), mixed (green), residential-low (red) and residential-middle (yellow).

#### 3.4.2. Noise Exposure Distribution Map for Bantama

**Figure 3:**
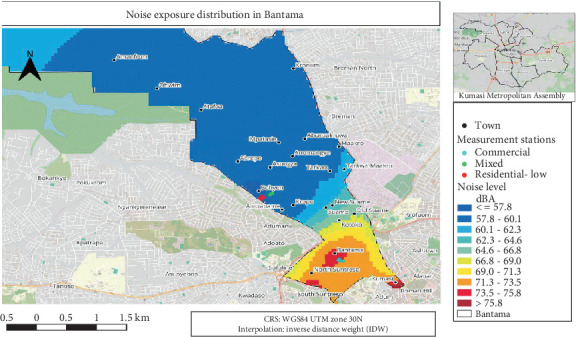
An average noise exposure distribution for Bantama.

Bantama neighbourhoods of the KMA depicted noise level spectrum ranged from ≤ 57.8 (blue) to > 75.8 dB (red), with the southern area surrounding Bantama and North Suntreso having the greatest levels (Figure 3). The northernmost areas, close to Ohwim and Amanfrom, were the quieter ones. Different land uses had various observation points labelled: commercial (sea blue), mixed (green) and residential-low (red).

#### 3.4.3. Noise Exposure Distribution Map for Nhyiaeso

**Figure 4:**
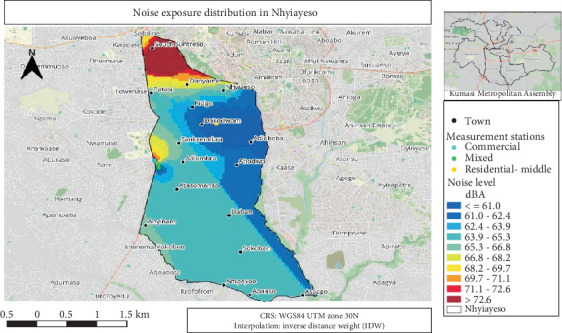
An average noise exposure distribution for Nhyiaeso.

Nhyiaeso noise levels ranged from ≤ 61 to > 72.6 dB, with higher exposure in South Suntreso and Patasi (Figure 4). The colour scale transitions from blue (lower noise) to red (higher noise). The map differentiates between residential-middle, mixed and commercial areas, with measurement stations marked by yellow, green and sea blue dots, respectively.

#### 3.4.4. Noise Exposure Distribution Map for Manhyia North and Manhyia South

**Figure 5:**
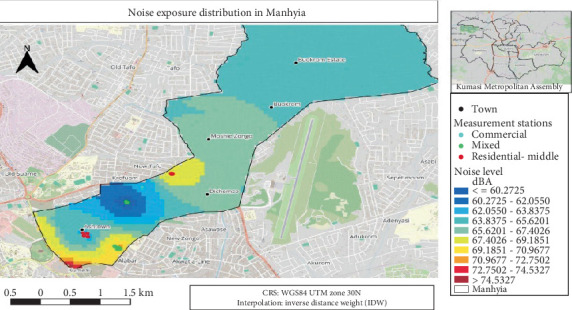
An average noise exposure distribution in Manhyia North and Manhyia South.

Figure 5 showed noise exposure levels in Manhyia, located in KMA. Noise levels were represented in dB (A) and varied across regions, with the highest exposure (> 74.5 dB) in the Ashanti New Town (Ashtown) area. The colour, from blue to red, indicated increasing noise levels. Measurement stations were marked with green, red and sea blue colour dots.

## 4. Conclusion and Recommendations

The average noise levels across five submetropolitan areas, including commercial and mixed areas, are above permissible limits. Commercial areas in KMA often exceed EPA permitted ambient noise levels, especially during peak afternoons. High traffic volumes, excessive honking, loud public address systems and dense human activity contribute to these high levels. Lower income communities have greater noise levels due to proximity to noise sources. Mixed neighbourhoods with a large commercial presence are more prone to noise pollution. The WHO's 2018 recommended limits for environmental noise, especially from road traffic, were continuously exceeded by the average noise levels in the Kumasi Metropolis across all submetros. Significant risks to health, such as cardiovascular problems, sleep disturbances, and cognitive impairment, are associated with this increased exposure. In order to completely understand the public health implications, more research including community-based assessments and effective noise mitigation techniques is urgently needed, as evidenced by the widespread exceedance. Deafening noise from public address systems should also be monitored and restricted, as should excessively honking from automobiles, under the oversight of the EPA Ghana.

## Figures and Tables

**Table 1:** Summary of WHO 2018 Environmental Noise Guidelines on recommended exposure limits and strength of recommendations for common environmental noise sources.

**Noise source**	**L**_**d** **e** **n**_ ** (dB)** ^**1**^	**L**_**n** **i** **g** **h** **t**_ ** (dB)** ^**1**^	**Recommendation** s**trength**^**2**^
Road traffic	< 53	< 45	Strong
Railway	< 54	< 44	Strong
Aircraft	< 45	< 40	Strong
Wind turbine	< 45	No recommendation^3^	Conditional
Leisure	Reducing the yearly average from all leisure noise sources combined to 70 dB *L*_Aeq,24h_^1^		Conditional

^1^ *L*_den_ is the day, evening and night sound level. It is the average sound level over a 24-h period, determined from the *L*_day_ (*L*_Aeq,12hr_ 7 am–7 pm), *L*_evening_ (*L*_Aeq,4hr_ 7 pm–11 pm) and *L*_night_ (*L*_Aeq,8hr_ 11 pm–7 am), with a 5-dB penalty added to the *L*_evening_ and a 10-dB penalty added to the *L*_night_. The *L*_Aeq,24hr_ is the average sound level over a 24-h period.

^2^Recommendations are rated as either strong or conditional. A strong recommendation *“…is based on the confidence that the desirable effects of adherence to the recommendation outweigh the undesirable consequences. The quality of evidence for a net benefit – combined with information about the values, preferences and resources—inform this recommendation…*”. For conditional recommendations, *“…There is less certainty of its efficacy owing to lower quality of evidence of a net benefit, opposing values and preferences of individuals and populations affected or the high resource implications of the recommendation, meaning there may be circumstances or settings in which it will not apply*”.

^3^The quality of evidence of night-time noise exposure to wind turbine noise is too low to allow recommendations.

**Table 2:** Ambient average noise of location and time in Subin.

**Location**	**Mean**	**Temp. (°C)**	**Humidity (%)**	**Std. deviation**	**Minimum**	**Maximum**
Morning						
Residential (middle)	56.94	27	85	11.80	46.00	69.85
Residential (low)	60.83	27	85	4.69	58.25	67.85
Mixed	62.90	27	85	5.13	55.80	67.30
Commercial	79.98	30	85	8.27	55.80	90.75
Afternoon						
Residential (middle)	61.28	31	49	2.09	59.75	64.25
Residential (low)	62.84	31	49	0.19	55.25	76.00
Mixed	67.74	31	48	4.41	62.05	72.75
Commercial	80.59	31	49	4.56	76.85	86.60
Evening						
Residential (middle)	60.01	28	81	2.53	57.1	62.9
Residential (low)	64.08	28	81	3.62	60.3	68.2
Mixed	59.80	28	81	1.96	57.95	62.15
Commercial	70.08	30	65	2.87	65.8	71.95

**Table 3:** Ambient average noise of location and time in Bantama.

**Location**	**Mean**	**Temp. (°C)**	**Humidity (%)**	**Std. deviation**	**Minimum**	**Maximum**
Morning						
Residential (low)	49.69	32	58	6.17	43.60	56.75
Mixed	61.30	33	56	2.74	59.80	65.40
Commercial	72.00	29	70	5.98	65.85	79.55
Afternoon						
Residential (low)	60.94	34	44	12.41	50.50	78.35
Mixed	80.33	35	41	4.11	54.95	64.65
Commercial	80.33	34	43	10.68	69.15	93.35
Evening						
Residential (low)	60.60	26	82	7.35	52.95	69.85
Mixed	53.20	26	82	4.91	48.75	59.65
Commercial	68.39	26	82	5.61	61.85	75.30

**Table 4:** Ambient average of noise of location and time in Manhyia North.

**Location**	**Mean**	**Temp. (°C)**	**Humidity (%)**	**Std. deviation**	**Minimum**	**Maximum**
Morning						
Residential (middle)	68.65	31	50	5.12	61.20	72.10
Mixed	60.15	33	44	3.38	56.65	64.35
Commercial	64.63	33	44	8.92	52.35	72.55
Afternoon						
Residential (middle)	68.51	34	41	3.37	64.25	72.50
Mixed	64.11	34	40	4.26	59.35	69.55
Commercial	59.95	34	40	2.29	57.60	62.80
Evening						
Residential (middle)	70.36	24	88	4.96	63.85	75.35
Mixed	54.76	23	94	3.73	50.95	59.90
Commercial	65.74	24	88	6.10	59.95	74.10

**Table 5:** Ambient average noise of location and time in Manhyia South.

**Location**	**Mean**	**Temp. (°C)**	**Humidity (%)**	**Std. deviation**	**Minimum**	**Maximum**
Morning						
Residential (middle)	64.13	32	58	3.92	58.25	66.20
Residential (low)						
Mixed	66.43	32	58	0.56	66.05	67.25
Commercial						
Afternoon						
Residential (middle)	67.84	31	54	7.20	63.10	78.50
Residential (low)						
Mixed	70.43	31	54	5.61	63.20	76.90
Commercial						
Evening						
Residential (middle)	56.89	26	82	6.46	51.60	65.80
Residential (low)						
Mixed	72.68	26	82	6.38	67.65	81.10
Commercial						

**Table 6:** Ambient average noise of location and time in Nhyiaeso.

**Location**	**Mean**	**Temp. (°C)**	**Humidity (%)**	**Std. deviation**	**Minimum**	**Maximum**
Morning						
Residential (middle)	57.20	26	86	4.67	53.90	64.10
Residential (low)						
Mixed	70.98	34	47	4.10	65.70	75.70
Commercial	71.85	34	47	2.73	68.70	75.35
Afternoon						
Residential (middle)	63.09	36	36	4.31	57.90	66.95
Residential (low)						
Mixed	57.96	36	37	4.85	54.35	65.00
Commercial	72.50	36	36	6.20	61.35	75.55
Evening						
Residential (middle)	59.78	30	56	4.07	54.74	64.60
Residential (low)						
Mixed	51.86	29	60	2.27	49.00	54.30
Commercial	74.83	29	60	8.51	69.05	87.35

**Table 7:** A measure of noise dose, duration of exposure and average noise level of all submetros.

**Submetro**	**Time**	**Average noise level (dB)**
Subin	Morning	70.10
	Afternoon	72.27
	Evening	65.69

Bantama	Morning	56.30
	Afternoon	62.16
	Evening	60.73

Manhyia North	Morning	59.52
	Afternoon	64.19
	Evening	63.62

Manhyia South	Morning	65.28
	Afternoon	69.13
	Evening	64.78

Nhyiaeso	Morning	66.68
	Afternoon	63.75
	Evening	62.16

**Table 8:** Regression analysis for ambient noise level, humidity, temperature and elevation.

**Model**	**Beta**	***Sig.***
(constant)	−22.267	*0.232*
Tempt	0.956	*0.000*
Humidity	0.085	*0.135*
Elevation	0.175	*0.000*

*Note:* Dependent variable: noise level. Significance is *p* < 0.05.

## Data Availability

Data is available upon request from the author.
